# Effects of Hyperlipidemia on the Pharmacokinetics of Tofacitinib, a JAK 1/3 Inhibitor, in Rats

**DOI:** 10.3390/pharmaceutics15092195

**Published:** 2023-08-24

**Authors:** Jong Mun Won, Hyeon Gyeom Choi, So Yeon Park, Jang-Hee Kim, So Hee Kim

**Affiliations:** 1 College of Pharmacy and Research Institute of Pharmaceutical Science and Technology, Ajou University, Suwon 16499, Republic of Korea; ddong7930@ajou.ac.kr (J.M.W.); ggb1212@ajou.ac.kr (H.G.C.); 2 Department of Biohealth Regulatory Science, Graduate School of Ajou University, Suwon 16499, Republic of Korea; qkrth0413@ajou.ac.kr; 3 Department of Pathology, Ajou University School of Medicine, Suwon 16499, Republic of Korea; drjhk@ajou.ac.kr

**Keywords:** tofacitinib, hyperlipidemia, poloxamer 407, CYP3A1/2, CYP2C11, P-glycoprotein, pharmacokinetics

## Abstract

Tofacitinib, an inhibitor of Janus kinases (JAKs) 1 and 3, has been shown to be effective in the treatment of rheumatoid arthritis. The incidence of hyperlipidemia has been found to be higher in patients with rheumatoid arthritis. The present study therefore investigated the pharmacokinetics of tofacitinib after its intravenous (10 mg/kg) or oral (20 mg/kg) administration in poloxamer-407-induced hyperlipidemic (PHL) rats. The area under the plasma concentration-time curve from zero to infinity (AUC_0–∞_) after intravenous administration of tofacitinib was 73.5% higher in PHL than in control rats, owing to slower time-averaged nonrenal clearance (CL_NR_) in the former. Evaluation of in vitro metabolism showed that the intrinsic clearance (CL_int_) of tofacitinib was 38.6% lower in PHL than in control rats, owing to the decreased protein expression of hepatic cytochrome P450 (CYP) 3A1/2 and CYP2C11 in PHL rats. Similar results were observed in PHL rats after oral administration of tofacitinib. These results were likely due to the decreased CL_NR_, CL_int_, and P-glycoprotein (P-gp) expression in the intestines of PHL compared to control rats. Overall, these findings indicated that hyperlipidemia slowed the metabolism of tofacitinib, increasing its plasma concentrations, and that this reduced metabolism was due to alterations in expression of the proteins CYP3A1/2, CYP2C11, and P-gp in the liver and/or intestines of PHL rats.

## 1. Introduction

**Figure 1:**
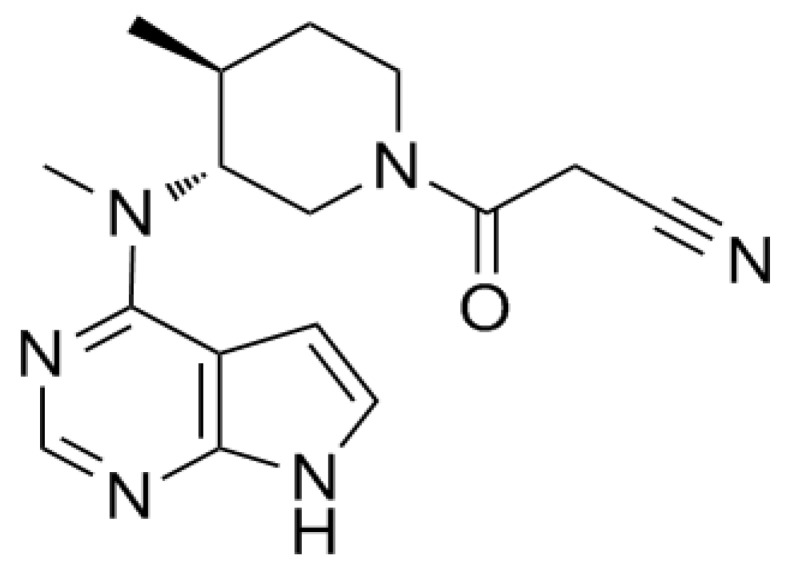
Structure of tofacitinib.

Tofacitinib (Figure 1) is an inhibitor of Janus kinases (JAKs) 1 and 3 [1,2] that has been shown to block receptors for interleukins-2, -4, -7, -9, -15, and -21, thereby modulating immune responses [3,4]. Tofacitinib has been shown to be effective in the treatment of several autoimmune diseases, including rheumatic and psoriatic arthritis, particularly in patients intolerant to methotrexate therapy [1,5]. Tofacitinib has also been approved for the treatment of moderate to severe ulcerative colitis and is the first oral JAK inhibitor approved for long-term use in these patients [5].

Tofacitinib is rapidly absorbed and eliminated, with a terminal half-life of approximately 3.3 h [6,7,8]. In humans, tofacitinib is primarily metabolized by cytochrome P450 (CYP) 3A4, followed by CYP2C19 [6]; thus, approximately 70% of the administered dose is eliminated non-renally, whereas 30% is excreted through the kidneys [6,7,8]. The pharmacokinetics of tofacitinib have also been investigated in patients with various diseases, including ankylosing spondylitis [9] and psoriasis [10], as well as in renal transplant recipients [11].

Chronic inflammation owing to rheumatoid arthritis promotes the secretion of inflammatory cytokines in immune cells, activates inducible nitric oxygen synthase (iNOS), and increases the release of free radical oxygen species, resulting in oxidative stress and abnormal lipid metabolism [12,13]. This can result in hyperlipidemia, a condition characterized by abnormally high blood levels of total cholesterol and/or triglycerides that are associated with hypertension, arteriosclerosis, and myocardial infarction [14,15].

Hyperlipidemia is known to significantly affect the pharmacokinetics of many drugs. Significantly slower rates of elimination of tadalafil [16], nifedipine [17], atazanavir [18,19], docetaxel [20], carbamazepine [21,22], and verapamil [23,24] were observed in hyperlipidemic rats owing to the decrease in the unbound fraction in plasma and/or intrinsic hepatic metabolism [25]. Although hyperlipidemia is frequently observed in patients with rheumatoid arthritis, less is known about the effects of hyperlipidemia on the pharmacokinetic of tofacitinib.

The present study was designed to assess the changes in the pharmacokinetics of orally and intravenously administered tofacitinib in a rat model of hyperlipidemia induced by poloxamer 407 (P-407). This study also evaluated the effects of hyperlipidemia on the in vitro metabolism of tofacitinib and the expression of CYP isoforms and P-glycoprotein (P-gp) in hepatic and intestinal microsomes.

## 2. Materials and Methods

### 2.1. Materials

Tofacitinib; hydrocortisone, an internal standard (IS) used for the quantification of tofacitinib; and P-407 were obtained from Sigma-Aldrich (St. Louis, MO, USA). A nicotinamide adenine dinucleotide phosphate hydrogen (NADPH)-generating system was obtained from Corning Inc. (Corning, NY, USA). β-Cyclodextrin was from Wako (Osaka, Japan); high-performance liquid chromatography (HPLC)-grade ethyl acetate and acetonitrile were from J.T. Baker (Phillipsburg, NJ, USA); and injectable 0.9% NaCl and heparin were from JW Pharmaceutical Co. (Seoul, Republic of Korea). Antibodies against CYP3A1/2, CYP2C11, CYP2D1, CYP2B1/2, and CYP1A1/2 were kindly donated by Detroit R&D Inc. (Detroit, MI, USA), and primary antibody against P-gp was purchased from Abcam (Cambridge, UK). β-Actin was obtained from Cell Signaling Technology (Beverly, MA, USA), and secondary goat, rabbit, and mouse antibodies were from Bio-Rad (Hercules, CA, USA). Other chemicals and solvents were of HPLC or analytical grade and were used without further purification.

### 2.2. Animals

Male Sprague–Dawley rats, aged 8 weeks and weighing 260–280 g, were obtained from OrientBio (Seongnam, Republic of Korea). The rats were individually housed in a clean room maintained at 21–23 °C under light (07:00–19:00) and dark (19:00–07:00) cycles of 12 h each and at a relative humidity of 45–55% through air purification (Laboratory Animal Research Center of Ajou University Medical Center, Suwon, Republic of Korea). The rats were treated as described previously [26,27]. All rats were provided free access to food and water during the entire experiment. The animal experiments and protocols were based on standard procedures and were approved by the Institutional Animal Care and Use Committee of Ajou University Medical Center (IACUC No. 2019-0021).

### 2.3. Induction of Hyperlipidemia

The rats were randomly assigned to one of two experimental groups: a control (CON) and a P-407-induced hyperlipidemia (PHL) group [28,29]. P-407 was dissolved in cold sterile 0.9% NaCl-injectable solution and stored in a refrigerator for at least 6 h to ensure complete dissolution. Rats in the PHL group received a single intraperitoneal injection of 1 g/kg P-407, whereas rats in the CON group received a single intraperitoneal injection of an equal volume of 0.9% NaCl. Hyperlipidemia was defined as a total cholesterol concentration higher than 220 mg/dL or a low-density lipoprotein (LDL) cholesterol concentration higher than 160 mg/dL [29,30].

### 2.4. Preliminary Study

Plasma concentrations of total cholesterol, LDL cholesterol, high-density lipoprotein (HDL) cholesterol, triglyceride, blood urea nitrogen (BUN), glutamate pyruvate transaminase (GPT), and glutamate oxaloacetate transaminase (GOT) were measured in CON and PHL rats (Green Cross Reference Lab, Seoul, Republic of Korea). To evaluate creatinine clearance (CL_CR_), urine samples were collected for 24 h and urine volumes and creatinine concentrations were measured, with CL_CR_ estimated by dividing the amount of creatinine excreted in the urine over 24 h by the area under the plasma concentration–time curve of creatinine from 0 to 24 h (AUC_0–24 h_) [26]. In addition, whole kidneys and livers were removed from CON and PHL rats and weighed, and biopsy samples were fixed in 10% neutral buffered formalin (BBC Biochemical, Mount Vernon, WA, USA).

### 2.5. Plasma Protein Binding of Tofacitinib

Plasma protein binding of tofacitinib obtained from three CON and PHL rats each was measured by ultrafiltration, similar to previously published methods [31]. Briefly, 0.5 mL fresh rat plasma containing 5 µg/mL tofacitinib was loaded into the reservoir of a Nanosep 10 K centrifugal filter device (Pall Co., Ann Arbor, MI, USA) and the samples were centrifuged at 1500× *g* for 30 min. The ultrafiltrate containing free tofacitinib was collected and a 100 µL aliquot of the ultrafiltrate was stored at −80 °C until HPLC analysis [32]. Nonspecific binding of tofacitinib to the ultrafiltration device was evaluated using phosphate buffer (pH 7.4) containing an equal concentration of tofacitinib (*n* = 3).

The binding percentage (%) was calculated using the equation:

$$\mathrm{Binding}\;\mathrm{percentage}\;\left(\%\right)=\frac{C_{T}-C_{F}}{C_{T}}\times 100$$

where *C_F_* represents the concentration of free drug measured in the ultrafiltrate and *C_T_* is the total drug concentration introduced into the ultrafiltration system before centrifugation [31].

### 2.6. Intravenous and Oral Administration of Tofacitinib

Rats were anesthetized with ketamine (100 mg/kg) prior to surgical procedures. Before intravenous administration of tofacitinib, the jugular vein of each rat was cannulated using polyethylene tubing 50 (Clay Adams, Parsippany, NJ, USA) for drug administration and the carotid artery was cannulated for blood collection. Prior to oral investigation, the rats were fasted overnight and only the carotid artery was cannulated for blood collection. The rats were allowed to recover for 3–4 h after surgical procedures prior to the performance of subsequent experiments [26,27].

Tofacitinib, dissolved in 0.9% NaCl containing 0.5% β-cyclodextrin, was administered intravenously through the jugular veins of CON (*n =* 7) and PHL (*n* = 6) rats at a dose of 10 mg/kg. Blood samples (0.12 mL) were obtained at 0 (prior to drug administration), 1, 5, 15, 30, 45, 60, 90, 120, 180, and 240 min through the carotid artery. Tofacitinib dissolved in the same solution was also orally administered to CON (*n* = 6) and PHL (*n* = 7) rats at a dose of 20 mg/kg. Blood samples (0.12 mL) were collected through the carotid artery at 0 (prior to drug administration), 5, 15, 30, 45, 60, 90, 120, 180, 240, 360, and 480 min. All blood samples were centrifuged for 1 min at 8000× *g* and plasma (50 μL) samples were collected [26,33]. After the blood samples were obtained, 0.3 mL of heparinized 0.9% NaCl solution (20 IU/mL) was immediately injected into the carotid artery to prevent blood clotting.

At 24 h, the rats were sacrificed and the abdomen of each was opened. The entire gastrointestinal tract of each rat was excised, cut into small pieces, and immersed in a beaker containing 50 mL of methanol. After evenly mixing the contents in the beaker, a 50 μL aliquot of each supernatant was withdrawn [26,33]. The volumes of urine samples collected for 24 h after drug administration were measured and two 100 μL aliquots of each were collected [26,33]. All samples were stored at −80 °C until the HPLC analysis of tofacitinib [32].

### 2.7. Distribution of Tofacitinib in Tissues

The distribution of tofacitinib in rat tissues was assessed as previously described [26,33]. Approximately 30 min after the intravenous administration of tofacitinib (10 mg/kg) to CON and PHL rats (*n* = 3 each), the maximum possible volume of blood was collected from the carotid artery and immediately centrifuged to obtain plasma. Tissue samples, including fat, brain, kidney, heart, liver, large intestine, mesentery, lung, small intestine, muscle, stomach, and spleen, were removed from each rat, rinsed in phosphate-buffered saline (PBS, pH 7.4), and blotted dry with paper towels to remove any remaining blood. Approximately 1 g of each tissue was added to 4 volumes of homogenizing buffer, followed by homogenization (IKA Labortechnik, Staufen, Germany) and centrifugation at 8000× *g* for 10 min. An aliquot (50 μL) of each supernatant was withdrawn and stored at −80 °C until the HPLC analysis of tofacitinib [32].

### 2.8. Measurement of V_max_, K_m_, and CL_int_

Hepatic and intestinal microsomes were prepared as described previously [26]. Protein concentrations of hepatic and intestinal microsomes were measured using bicinchoninic acid (BCA) assays. Microsomes (1 mg protein), 1 μL of various concentrations of tofacitinib (0.5, 0.75, 1, 2, 5, 10, and 20 μM), and the NADPH-generating system were mixed to simulate an in vivo metabolic system, with the total volume of the system adjusted to 1 mL by adding 0.1 M PBS (pH 7.4). The contents were incubated at 50 oscillations per min (opm) in a water bath at 37 °C for 15 min and the reactions were terminated by adding twice the volume of methanol. Kinetic constants, maximum velocity (*V*_max_) and apparent Michaelis–Menten constant (*K*_m_; the concentration at which the rate is one half of *V*_max_ for the metabolism of tofacitinib) were measured using Lineweaver–Burk plots, followed by nonlinear regression analysis [26,34] using Prism 5 version 5.01 software (GraphPad Software Inc., San Diego, CA, USA). The intrinsic clearance (CL_int_) for tofacitinib metabolism was calculated by dividing *V*_max_ by *K*_m_ [26,34].

### 2.9. Immunoblot Analysis

Hepatic and intestinal microsomes (20–40 μg of protein per lane) were resolved by 10% sodium dodecyl sulfate polyacrylamide gel electrophoresis and transferred to a nitrocellulose membrane for 1 h. For immunodetection, the blots were incubated on a rotary shaker overnight at 4 °C with primary antibodies against CYP1A1/2, CYP2B1/2, CYP2C11, CYP2D1, CYP3A1/2, and P-gp diluted in Tris-buffered saline containing 0.1% Tween 20 (TBS-T) and 5% bovine serum albumin (1:2000). The blots were washed and incubated for 1 h at room temperature with a horseradish peroxide-conjugated secondary antibody diluted 1:10,000 in TBS-T containing 5% skim milk. Protein expression was visualized by enhanced chemiluminescence (Bio-Rad) using an Image Quant LAS 4000 Mini (GE Healthcare Life Sciences, Piscataway, NJ, USA) and the band density was measured using ImageJ 1.45 s software (NIH, Bethesda, MA, USA). β-Actin was used as a loading control [26].

### 2.10. HPLC Analysis

Tofacitinib concentrations in biological samples were quantified as described previously [32]. Briefly, 50 μL of each biological sample was mixed with 1 μL of 5 mg/mL hydrocortisone (an internal standard), followed by the addition of 20 μL of 20% ammonia solution. Each mixture was vortexed for 30 s and extracted with 750 μL of ethyl acetate. The organic layer was collected and evaporated under a gentle flow of nitrogen gas at 40 °C (Eyela, Tokyo, Japan). The residue was redissolved in 130 μL of 20% acetonitrile and 50 μL of the supernatant was loaded onto a reversed-phase column (C_18_; 250 × 4.6 mm, 5 μm; Young Jin Biochrom, Seongnam, Republic of Korea) and analyzed by HPLC.

The tofacitinib concentrations in the biological samples were determined using a Prominence LC-20A HPLC system (Shimadzu, Kyoto, Japan). The mobile phase consisted of a 69.5:30.5 (*v*/*v*) ratio of 10 mM ammonium acetate buffer (pH 5.0) and acetonitrile, with a flow rate of 1 mL/min. Concentrations of tofacitinib and hydrocortisone were measured using a UV detector set at 287 nm. The retention times of tofacitinib and hydrocortisone were approximately 7.21 and 11.3 min, respectively.

The lower limits of quantification of tofacitinib in rat plasma, urine, and tissues were 0.01, 0.1, and 0.1 μg/mL, respectively. The intraday assay precisions (coefficients of variation; CVs) in rat plasma, urine, and tissues were 3.69–5.5%, 4.21–6.18%, and 1.11–8.74%, respectively, and the corresponding interday assay precisions in rat plasma and urine were 5.06% and 5.46%, respectively [32].

### 2.11. Pharmacokinetic Analysis

The following pharmacokinetic parameters were determined by non-compartmental analysis (WinNonlin, Pharsight Co., Mountain View, CA, USA) using standard methods [35]: terminal half-life, time-averaged total body (CL), renal (CL_R_), and nonrenal (CL_NR_) clearances, apparent volume of distribution at steady state (*V*_ss_), area under the plasma concentration–time curve from zero to infinity (AUC_0–∞_), and elimination rate constant (*k*). AUC_0–∞_ was calculated using the trapezoidal rule extrapolation method [36], whereas the maximum plasma concentration (*C*_max_) and time required to reach *C*_max_ (*T*_max_) were determined directly from the plasma concentration–time curves. (R3-3) The absorption rate constants (*k*_a_) for oral administration were also estimated under the assumption of one-compartment model and first-order elimination.

### 2.12. Statistical Analysis

Most of the experimental results are presented as the mean ± standard deviation (SD), whereas *T*_max_ is presented as the median (range). Results in the CON and PHL groups were compared using unpaired Student’s *t*-tests, with *p* values < 0.05 considered statistically significant using Prism 5 version 5.01 software (GraphPad Software Inc., San Diego, CA, USA).

## 3. Results

### 3.1. Preliminary Study

**Figure 2:**
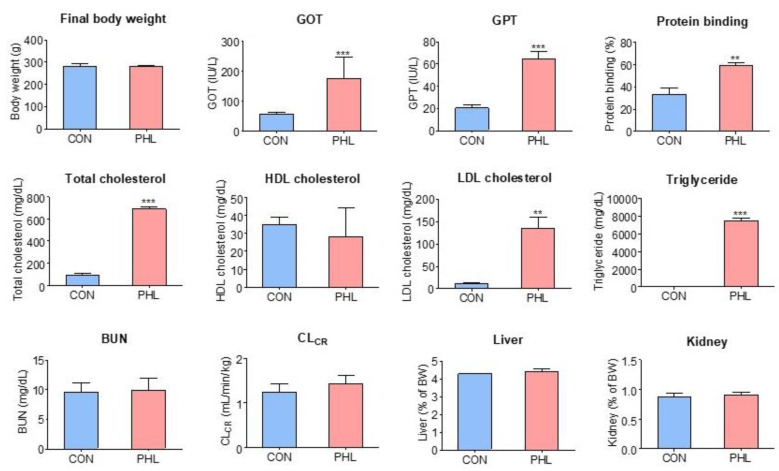
Mean (± standard deviation) final body weight; blood concentrations of BUN, GOT, GPT, triglycerides, LDL cholesterol, HDL cholesterol, and total cholesterol; CL_CR_; plasma protein binding; and relative liver and kidney weights in CON and PHL rats. BUN, blood urea nitrogen; GOT, glutamate oxaloacetate transaminase; GPT, glutamate pyruvate transaminase; CL_CR_, creatinine clearance; LDL, low-density lipoprotein; HDL, high-density lipoprotein; CON, control; PHL, poloxamer-407-induced hyperlipidemia. ** *p* < 0.01; *** *p* < 0.001 compared with CON rats.

Body weights, biochemical parameters, plasma protein binding of tofacitinib, CL_CR_, and relative liver and kidney weights were compared in CON and PHL rats (Figure 2). Their final body weights did not differ significantly, but PHL rats showed impaired liver function. GOT and GPT concentrations were 132% and 217% higher, respectively, in PHL than in CON rats, with both differences being statistically significant. Total cholesterol, LDL cholesterol, and triglyceride concentrations were also significantly higher in PHL than in CON rats by 635%, 1118%, and 8912%, respectively, whereas HDL cholesterol concentrations were comparable in CON and PHL rats. BUN and CL_CR_ concentrations were also comparable in CON and PHL rats. Moreover, their relative liver and kidney weights (% of body weight) did not differ significantly. No unusual morphological findings or histologically significant changes were observed in the livers and kidneys of PHL rats.

### 3.2. Plasma Protein Binding of Tofacitinib

The plasma protein binding values of tofacitinib were 33.3 ± 6.05% and 59.2 ± 2.49% for CON and PHL rats (*n* = 3, each), respectively, and were significantly higher (*p* < 0.001) in PHL rats than in CON rats (Figure 2). After the ultrafiltration of phosphate buffer (pH 7.4) containing tofacitinib (5 µg/mL), the mean recovery was 100 ± 2.84% (*n* = 3). This indicates negligible nonspecific binding of tofacitinib to the ultrafiltration device.

### 3.3. Pharmacokinetics of Tofacitinib following Intravenous and Oral Administration

**Figure 3:**
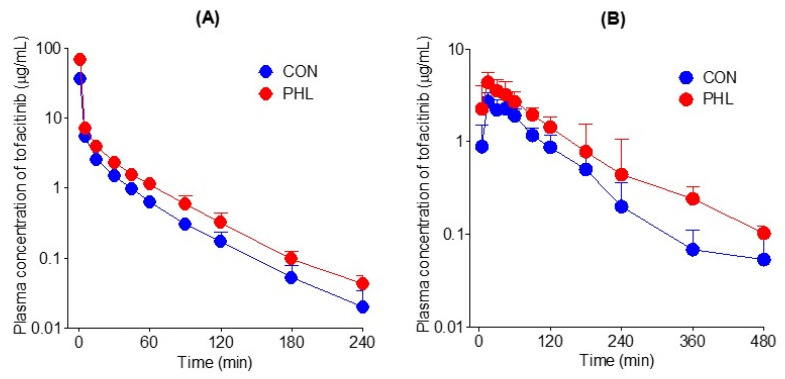
(**A**) Mean arterial plasma concentration–time curves of tofacitinib after intravenous administration of 10 mg/kg tofacitinib to CON (*n* = 7) and PHL (*n* = 6) rats. (**B**) Mean arterial plasma concentration–time curves of tofacitinib after oral administration of 20 mg/kg tofacitinib to CON (*n* = 6) and PHL (*n* = 7) rats. The bars represent standard deviations. CON, control; PHL, poloxamer-407-induced hyperlipidemia.

The mean arterial plasma concentration–time curves of tofacitinib were assessed after its intravenous administration at a dose of 10 mg/kg to CON and PHL rats (Figure 3A), with the relevant pharmacokinetic parameters of tofacitinib shown in Table 1. The mean arterial plasma concentrations of tofacitinib were higher in PHL than in CON rats, with the AUC_0–∞_ being 73.5% higher in PHL rats. CL and CL_NR_ were significantly slower by 43.0% and 62.4%, respectively, in PHL than in CON rats. In contrast, *V*_ss_ was 43.3% lower in PHL rats than in CON rats, likely because plasma protein binding of tofacitinib was significantly higher in PHL rats. The percentage of tofacitinib excreted unchanged in urine for 24 h (*Ae*_0–24 h_) and the terminal half-life of tofacitinib did not differ significantly in CON and PHL rats.

The mean arterial plasma concentration–time profiles of tofacitinib were also assessed after its oral administration at a dose of 20 mg/kg to CON and PHL rats (Figure 3B), with several of the relevant pharmacokinetic parameters of tofacitinib shown in Table 1. Orally administered tofacitinib was rapidly absorbed in the gastrointestinal tract, being detected in plasma 5 min after dosing in all CON and PHL rats. Mean arterial plasma concentrations of tofacitinib were higher in PHL than in CON rats, with the AUC_0–∞_ and *C*_max_ of the drug being 65.6% and 60.5% higher, respectively, in PHL than in CON rats. However, terminal half-life and *k*_a_ values were comparable in CON and PHL rats. CL_R_ value was significantly lower, by 63.6%, in PHL than in CON rats, owing to the significantly higher AUC_0–∞_ in PHL rats. The absolute bioavailability (*F*) of tofacitinib after oral administration was comparable in CON and PHL rats, at 53.7% and 51.4%, respectively.

### 3.4. Distribution of Tofacitinib in Tissues

**Figure 4:**
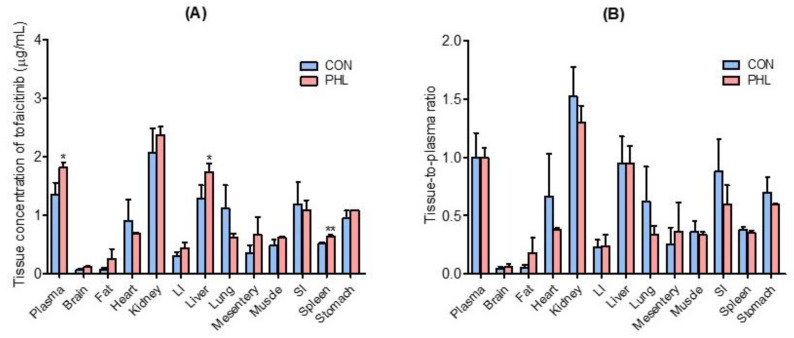
Mean ± standard deviation (**A**) tissue (μg/g) and plasma (μg/mL) concentrations and (**B**) tissue-to-plasma (T/P) ratios of tofacitinib 30 min after intravenous administration of 10 mg/kg to CON and PHL rats (*n* = 3 each). Bars represent standard deviations. Abbreviations: LI, large intestine; SI, small intestine; CON, control; PHL, poloxamer 407-induced hyperlipidemia. * *p* < 0.05 and ** *p* < 0.01 compared with CON rats.

Tissue and plasma concentrations of tofacitinib, along with tissue-to-plasma (T/P) ratios, were measured 30 min after intravenous administration of 10 mg/kg of tofacitinib to CON and PHL rats (Figure 4). Tofacitinib was widely distributed in both groups, and their plasma and tissue concentrations of tofacitinib were generally comparable, except for the liver and spleen (Figure 4A). The T/P ratios of tofacitinib were lower than 1.0 in all tissues of CON and PHL rats (Figure 4B). This may have been due to the lower *V*_ss_ in both CON (847 mL/kg) and PHL (490 mL/kg) rats (Table 1).

### 3.5. Measurement of K_m_, V_max_, and CL_int_ of Tofacitinib

**Figure 5:**
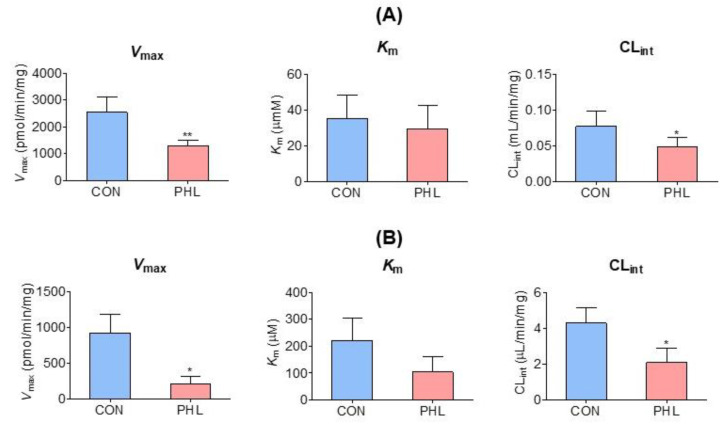
Mean (± standard deviation) *V*_max_, *K*_m_, and CL_int_ for the disappearance of tofacitinib in (**A**) hepatic and (**B**) intestinal microsomes obtained from CON and PHL rats (*n* = 3, each). The experiments were repeated three times. Bars represent standard deviations. *V*_max_, maximum velocity; *K*_m_, tofacitinib concentration at one-half *V*_max_; CL_int_, intrinsic clearance; CON, control; PHL, poloxamer-407-induced hyperlipidemia. * *p* < 0.05 and ** *p* < 0.01 compared with the CON group.

*V*_max_, *K*_m_, and CL_int_ for the disappearance of tofacitinib were also evaluated in the hepatic and intestinal microsomes of CON and PHL rats (Figure 5). Although *V*_max_ and CL_int_ for the disappearance of tofacitinib were significantly lower by 48.4% and 36.9%, respectively, in the hepatic microsomes of PHL than of CON rats, *K*_m_ did not differ significantly in these groups (Figure 5A). Similar results were observed in the intestinal microsomes of these rats, in that *V*_max_ and CL_int_ were significantly lower by 77.5% and 51.3%, respectively, in PHL than in CON rats, and *K*_m_ for tofacitinib was comparable in these two groups (Figure 5B). These findings suggested that the in vitro metabolism of tofacitinib in rat liver and intestine was affected by hyperlipidemia.

### 3.6. Protein Expression of CYP Isozymes

**Figure 6:**
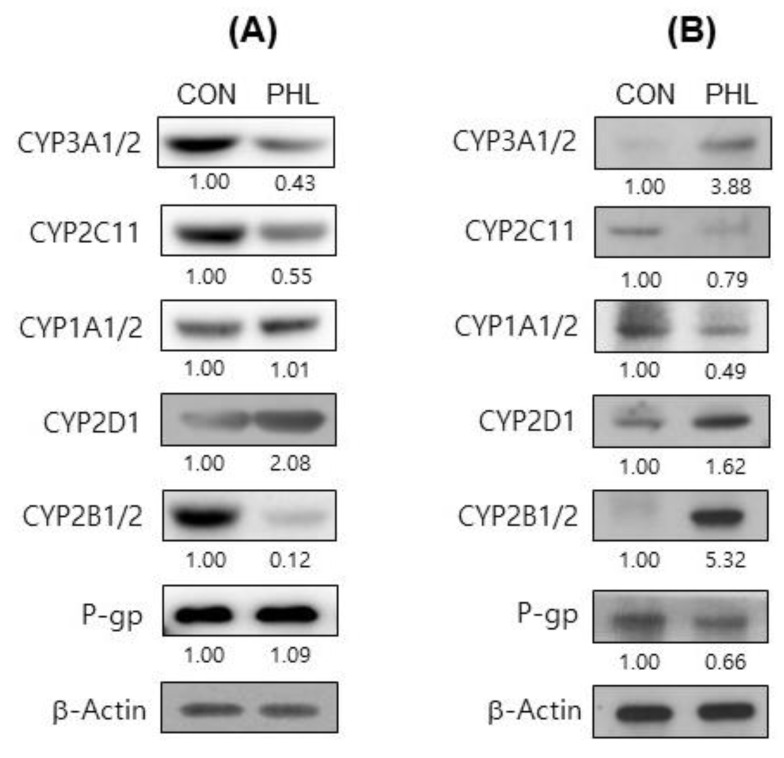
Immunoblot analyses of CYP isozymes and P-gp in (**A**) hepatic and (**B**) intestinal microsomes obtained from CON and PHL rats. β-Actin was used for the loading control. Results shown are representative of three identical experiments. Band density was determined by ImageJ 1.45s software (NIH). CYP, cytochrome P450; P-gp, P-glycoprotein; CON, control; PHL, poloxamer-407-induced hyperlipidemia.

To further assess the mechanism by which hyperlipidemia affects tofacitinib metabolism, the protein expression of CYP isozymes in rat hepatic and intestinal microsomes was evaluated (Figure 6). The levels of CYP2B1/2, CYP3A1/2, and CYP2C11 proteins were considerably lower and the level of CYP2D6 was higher in the hepatic microsomes of PHL than of CON rats, whereas CYP1A1/2 levels were comparable in the two groups (Figure 6A). In intestinal microsomes, however, the levels of expression of CYP2B1/2, CYP2D6, and CYP3A1/2 were higher, whereas the levels of expression of CYP1A1/2 and CYP2C11 were lower in PHL than in CON rats (Figure 6B). The protein expression level of P-gp was lower in the intestinal microsomes of PHL than of CON rats, whereas the levels of P-gp were comparable in the hepatic microsomes of these rats. These findings suggested that hyperlipidemia affected the expression of P-gp in the intestine, possibly resulting in the increased absorption of tofacitinib observed in the intestines of PHL rats.

## 4. Discussion

P-407 is a nonionic surfactant that has been found to inhibit lipoprotein lipase, an enzyme involved in the hydrolysis of triglycerides, and to enhance the activity of 3-hydroxy-3-methyl-glutaryl-coenzyme A (HMG-CoA) reductase, resulting in acute hyperlipidemia that can cause atherosclerosis [37,38,39]. P-407 was found to rapidly induce hyperlipidemia, with this induction being highly reproducible and nontoxic [40]. The present study found that alterations in lipid metabolism, owing to the reduced hydrolysis of triglycerides, increased the levels of triglycerides, LDL cholesterol, and total cholesterol in PHL rats. Although no morphological or histological changes were observed in the livers of PHL rats, GOT and GPT levels were significantly higher than in CON rats, owing to the induction of hyperlipidemia. These findings suggest that hepatic damage is a likely consequence of hyperlipidemia, thus altering drug metabolism. However, BUN and CL_CR_ were comparable in CON and PHL rats, suggesting that hyperlipidemic conditions did not alter the renal function.

The AUCs of tofacitinib intravenously (5–50 mg/kg) and orally (10–100 mg/kg) administered to male Sprague–Dawley rats were previously shown to be dose-dependent at doses higher than 50 mg/kg for both routes of administration [33]. Thus, in the present study, the intravenous and oral doses of tofacitinib were set at 10 and 20 mg/kg, respectively.

The significantly higher AUC_0–∞_ observed in PHL compared to CON rats following the intravenous administration of tofacitinib may have been due to CL being significantly slower in PHL than in CON rats. The slower CL in PHL rats was likely due to CL_NR_ being significantly slower in PHL than in CON rats. The contribution of gastrointestinal (including biliary) excretion of unchanged tofacitinib to CL_NR_ was almost negligible [33]. Notably, the percentage of administered tofacitinib recovered from the gastrointestinal tract at 24 h (GI_24 h_) was below the limit of detection for both CON and PHL rats. Tofacitinib at a concentration of 5 μg/mL was found to be stable at 37 °C for 24 h in buffer solutions at pH values of 2, 4, 7, 9, and 10 [32]. Moreover, the biliary excretion of unchanged tofacitinib over 24 h was almost negligible (0.703 ± 0.303% of the dose) after its intravenous administration at 10 mg/kg [33]. Taken together, these findings suggest that the CL_NR_ of tofacitinib could represent its metabolic clearance, with the changes in CL_NR_ observed in this study indicating alterations in its hepatic metabolism in rats.

The hepatic clearance of drugs metabolized by hepatic CYPs and having a low to intermediate hepatic extraction ratio has been shown to be sensitive to CL_int_ and the unbound fraction (f_u_) of drugs [25]. Tofacitinib has an intermediate extraction ratio of 42% [33]. The slower CL_NR_ of tofacitinib in PHL compared to in CON rats may have been due to its significantly slower in vitro hepatic CL_int_ and decreased f_u_. The CL and/or CL_NR_ of nifedipine [17], atazanavir [18], docetaxel [41], amiodarone [42], and nelfinavir [43] were found to be lower in PHL than in CON rats. The increased plasma protein binding of tofacitinib reduced its free fraction in PHL rats, resulting in a significantly reduced *V*_ss_. Reduced *V*_ss_ coupled with increased plasma protein binding in PHL rats has also been reported for other drugs, including atazanavir [18], docetaxel [41], amiodarone [42], nelfinavir [43], and cyclosporine A [44]. Substantial increases in the plasma concentrations of amiodarone [42], atazanavir [18], docetaxel [41], and nelfinavir [43] and decreases in their clearance, volume of distribution, and unbound fraction have also been observed in hyperlipidemic rats.

The hepatic first-pass effect of tofacitinib after absorption into the portal vein was 42.0% in rats [33], indicating that this drug has an intermediate (30–70%) hepatic extraction ratio. Therefore, the lower hepatic CL_int_ in PHL rats was regarded as mainly due to a decrease in protein expression, with the levels of hepatic CYP2C11 and CYP3A1/2 being 45% and 57% lower, respectively, in PHL than in CON rats. The downregulation of hepatic CYP3A1/2 and CYP2C11 (by 38.4–51.9%) in hyperlipidemic rats [42] has been associated with decreases in the in vitro CL_int_ of amiodarone [42], carbamazepine [45], verapamil [28], and tolbutamide [29].

Following its intravenous administration, the CL_R_ values of tofacitinib could be estimated from the free (i.e., unbound to plasma proteins) fraction of the drug in plasma, which was found to be 66.7% in CON rats and 40.8% in PHL rats. The CL_R_ values of tofacitinib were, therefore, estimated to be 4.66 mL/min/kg for CON rats and 6.05 mL/min/kg for PHL rats. These values were considerably higher than the measured CL_CR_ values, which were 1.24 mL/min/kg in CON rats and 1.43 mL/min/kg in PHL rats, indicating that tofacitinib was actively secreted from the renal tubules of all rats [33]. Additionally, the similar BUN and CL_CR_ values in the two groups of rats indicated that renal function was not altered under hyperlipidemic conditions.

Following its oral administration, the AUC_0–∞_ of tofacitinib was also significantly higher in PHL than in CON rats, with the *F* values of tofacitinib being comparable in the two groups. Relative to its oral dose, the intestinal first-pass effect of tofacitinib was 46.1%, whereas its hepatic first-pass effect after absorption into the portal vein was 21.3% [33]. The significantly greater oral AUC_0–∞_ of tofacitinib in PHL than in CON rats may have been due primarily to reduced metabolism in the intestine followed by reduced metabolism in the liver. Based on the in vitro intestinal metabolism of tofacitinib, its *V*_max_ was significantly lower in PHL than in CON rats, with CL_int_ also being significantly lower in PHL rats. This may have been due, at least in part, to the considerably lower expression of CYP2C11 protein in the intestinal microsomes of PHL rats. In contrast to the liver, the expression of CYP3A1/2 protein increased in the intestines of PHL rats. The exact reasons are unknown but CYP3A activity and/or protein expression have been reported to increase in the intestine but decrease in the liver in rat models of cisplatin-induced renal failure [27,46]. However, the plasma concentration of tofacitinib increased after its oral administration, at a dose of 20 mg/kg, to rats with renal failure [27].

The present study also found that the expression of P-gp protein was lower in the intestines of PHL rats. Because tofacitinib is a substrate of P-gp [47], these findings indicated that the decreased expression of P-gp increased the absorption of tofacitinib in PHL rats and partly contributed to the increase in AUC_0–∞_ after oral administration of tofacitinib. Higher AUC_0–∞_ values were, therefore, attributed to the increased absorption and reduced metabolism of tofacitinib owing to the decreased expression of P-gp and CYP2C11 in the intestines of PHL rats. Higher AUC_0–∞_ values have also been observed in hyperlipidemic rats after oral administration of carbamazepine [21,22], nifedipine [17], and atazanavir [18,19].

The concentrations of tofacitinib were comparable in various tissues of CON and PHL rats. The T/P ratios of tofacitinib were lower than 1.0 in both groups and were also comparable. Significantly higher plasma concentrations of tofacitinib were not reflected by higher tissue concentrations in PHL rats. This may have been due to the plasma protein binding of tofacitinib being significantly greater in PHL rats, resulting in a significantly lower *V*_ss_. The plasma protein binding of drugs was shown to be increased in hyperlipidemic rats, thereby reducing the volume of distribution [20,48]. The increased concentration of lipoproteins observed in hyperlipidemic rats may provide a greater binding capacity to a number of lipophilic drugs, thereby increasing their binding to proteins [49]. Similar results have also been reported with other drugs, including cyclosporine A [44], atazanavir [18], halofantrine [50], docetaxel [41], amiodarone [42], and nelfinavir [43].

## 5. Conclusions

The CL_NR_ of intravenously administered tofacitinib was found to be significantly slower in PHL than in CON rats, due possibly to the slower hepatic CL_int_ in PHL rats. This, in turn, may have resulted from the lower hepatic CYP3A1/2 and CYP2C11 levels in PHL than in CON rats. The AUC_0–∞_ of orally administered tofacitinib was also significantly higher in PHL than in CON rats, due possibly to the slower intestinal CL_int_ in PHL rats. This, in turn, may have resulted from the decreased expression of CYP2C11 and the increased absorption of tofacitinib in PHL rats, which may have been due to the lower level of expression of P-gp in the intestines of PHL than of CON rats. These findings, showing that hyperlipidemic conditions alter the pharmacokinetics of tofacitinib, may provide useful information for the clinical application of tofacitinib to patients with rheumatoid arthritis and hyperlipidemia. Furthermore, these results may aid in adjusting tofacitinib dosages for these patients.

## Figures and Tables

**Table 1:** Mean (± standard deviation) pharmacokinetic parameters of tofacitinib after intravenous administration of 10 mg/kg tofacitinib or oral administration of 20 mg/kg tofacitinib to CON and PHL rats.

Parameters	Intravenous		Oral	
	CON (*n* = 7)	PHL (*n* = 6)	CON (*n* = 6)	PHL (*n* = 7)
Body weight (g)	296 ± 4.69	294 ± 11.4	235 ± 4.71	250 ± 17.9
Terminal half-life (min)	29.6 ± 4.55	34.5 ± 2.29	62.1 ± 16.7	84.3 ± 21.2
*k* (min^−1^)	0.0239 ± 0.00337	0.0202 ± 0.00131	0.0118 ± 0.00301	0.00870 ± 0.00233
AUC (µg × min/mL)	268 ± 34.4	465 ± 24.1 ***	288 ± 38.6	477 ± 44.6 ***
*k*_a_ (min^−1^)			0.127 ± 0.0389	0.156 ± 0.0885
*C*_max_ (µg/mL)			2.96 ± 0.643	4.75 ± 1.18 **
*T*_max_ (min)			25.0 ± 14.7	30.0 ± 25.4
CL (mL/min/kg)	37.9 ± 4.85	21.6 ± 1.13 ***		
CL_R_ (mL/min/kg)	3.11 ± 1.58	2.47 ± 1.10	3.52 ± 1.80	1.28 ± 0.786 *
CL_NR_ (mL/min/kg)	34.8 ± 4.55	18.4 ± 1.25 ***		
MRT (min)	22.6 ± 5.13	22.7 ± 5.88		
*V*_ss_ (mL/kg)	847 ± 191	490 ± 136 **		
*Ae*_0–24 h_ (% of dose)	4.06 ± 1.89	5.76 ± 2.53	4.90 ± 1.95	3.02 ± 1.87
*F* (%)			53.7	51.4

*Ae*_0–24 h_, drug excreted unchanged in urine for 24 h; AUC_0–∞_, area under the plasma concentration–time curve from zero to infinity; *C*_max_, maximum plasma concentration; CL, time-averaged total body clearance; CL_NR_, time-averaged nonrenal clearance; CL_R_, time-averaged renal clearance; *F*, absolute oral bioavailability; *k*, elimination rate constant; *k*_a_, absorption rate constant; MRT, mean residence time; *T*_max_, time required to reach *C*_max_; *V*_ss_, apparent volume of distribution at steady state; CON, control; PHL, poloxamer-407-induced hyperlipidemia. * *p* < 0.05; ** *p* < 0.01; *** *p* < 0.001 compared with CON rats.

## Data Availability

The data presented in this study are available in this article (and Supplementary Materials).

## Supplementary Materials

The following supporting information can be downloaded at: https://www.mdpi.com/article/10.3390/pharmaceutics15092195/s1.
